# A Rare Case of Aeromonas Hydrophila Catheter Related Sepsis in a Patient with Chronic Kidney Disease Receiving Steroids and Dialysis: A Case Report and Review of Aeromonas Infections in Chronic Kidney Disease Patients

**DOI:** 10.1155/2013/735194

**Published:** 2013-08-24

**Authors:** Muhammad Abdul Mabood Khalil, Abdur Rehman, Waqar Uddin Kashif, Manickam Rangasami, Jackson Tan

**Affiliations:** ^1^Section of Nephrology Department of Medicine, Aga Khan University Hospital, Karachi 74800, Pakistan; ^2^Department of Nephrology, Kuala Belait Hospital, Kuala Belait KA1131, Brunei Darussalam; ^3^Department of Nephrology, RIPAS Hospital, Bander Seri Begwan BA1710, Brunei Darussalam

## Abstract

Aeromonas hydrophila (AH) is an aquatic bacterium. We present a case of fifty-five-year-old gentleman with chronic kidney disease (CKD) due to crescentic IgA nephropathy who presented to us with fever. He was recently pulsed with methyl prednisolone followed by oral prednisolone and discharged on maintenance dialysis through a double lumen dialysis catheter. Blood culture from peripheral vein and double lumen dialysis catheter grew AH. We speculate low immunity due to steroids and uremia along with touch contamination of dialysis catheter by the patient or dialysis nurse could have led to this rare infection. Dialysis catheter related infection by AH is rare. We present our case here and take the opportunity to give a brief review of AH infections in CKD patients.

## 1. Introduction

 AH is Gram-negative, rod-shaped facultative anaerobe. It can exist in aquatic environment, fish, food, birds, pets, and natural soil. AH can cause gastrointestinal and Nongastrointestinal infections. Nongastrointestinal infections include hemolytic syndrome and kidney disease, cellulitis, wound and soft-tissue infection, meningitis, bacteremia and septicemia, ocular infections, pneumonia and respiratory tract infections, and urinary tract infection in neonates, osteomyelitis, peritonitis, and acute cholecystitis [1–4].

 AH can cause infection in immunocompromised host. Ko and his colleagues found AH sepsis in liver cirrhosis (54%) and malignancy (21%) [5]. AH sepsis has also been documented in other conditions which impair host immune defenses. These conditions include leukemias, lymphomas, myelodysplasia [6] diabetes mellitus, kidney dysfunction, cardiac anomalies, aplastic anemia, thalassemia, multiple myeloma, and waldenstrom macroglobulinemia [7–11].

## 2. Case Report

A fifty-five-year-old Afghanistan gentleman with a history of hyperthyroidism (5 years) and ischemic cardiomyopathy (2 years) presented to Aga Khan University Hospital in July 2012. He was found to have kidney failure 2 months previously and was initiated on hemodialysis due to uremic encephalopathy. He was admitted to our institution for further work-up investigations for his kidney failure. His laboratory investigations were as follows:

  hemoglobin: 9 g/dL,
  white cell counts: 4,700 u/L,
  platelet count: 171,000/mm^3^.

Urine DR showed yellow color with clear appearance, pH of 5, specific gravity of 1.010, 4 red blood cells per high-power field, 1 WBCs per high-power field, urine protein of 1.0 g/L, and 2 RBCs cast and

 urine protein to creatinine ratio: 3.9,
  BUN: 109 mg/dL,
  creatinine: 4.8 mg/dL,
  electrolytes: serum sodium 141 mmol/L, potassium 5.0 mmol/L, chloride 116 mmol/L, and bicarbonate 14.5 mmol/L,
  ANA: negative,
  C-ANCA: negative,
  P-ANCA: negative,
  hepatitis B surface antigen: nonreactive,
  hepatitis C antibody: nonreactive,
  PT/APTT/INR: 10.5/30.2/1.

Because of symptomatic uremia, a right internal jugular nontunneled catheter was inserted on 28 of July 2012 for hemodialysis. In view of his normal size kidneys, a biopsy was done on 1 August 2012 to look for reversible causes. The biopsy showed 20 glomeruli in which 14 were globally sclerosed or severely collapsed and condensed. Several of them were associated with fibrocellular crescents with disruption of bowman's capsule and 4 had cellular crescents. Immunofluorescence showed IgA positivity in the mesangium and along the capillary walls. He was pulsed with methyl prednisolone 500 mg once a day for 3 days followed by oral prednisolone 25 mg twice a day. Cyclophosphamide or mycophenolate mofetil was not added in view of 14/20 sclerosed glomeruli and fibrocellular crescents. As there was no sign of renal recovery, the patient was discharged on three times a week dialysis through right internal jugular catheter with followup in nephrology outpatients clinic.

He was readmitted on 8 August 2012 approximately one week after discharge, with a two-day history of high grade fever, mild productive cough, frothy sputum, and hemoptysis. On physical examination, he was febrile with a temperature of 39°C, a heart rate of 100 beats per minute, a respiratory rate of 24 breaths per minute, blood pressure of 139/68 mmHg, and oxygen saturation of 99% at room air. He was pale and nonedematous. He had bilateral basal crepitations on chest examination. The rest of the physical exam was unremarkable. His laboratory workup showed BUN 74 mg/dL, serum creatinine, 4.8 mg/dL, serum sodium 135 mmol/L, potassium 4.7 mmol/L, chloride 109 mmol/L, and bicarbonate 19.1 mmol/L. His CBC showed hemoglobin of 7.1 g/dL, hematocrit of 25%, MCV 82 fl, WBC 3000 u/L, and platelet count of 70000/mm^3^. There was no evidence of hemolysis as his reticulocyte count was 2% and LDH was 250 IU/L. His iron study showed trasferrin saturation of 12%. Chest X-ray showed bilateral hilar congestion and minimal bilateral pleural effusion. Blood cultures were sent both from peripheral vein and dialysis catheter. In view of one episode of hemoptysis, bronchoscopy was done which was normal. Bronchoalveolar lavage showed no growth of acid fast bacilli and fungal smear was negative. Blood cultures from peripheral veins showed AH which was sensitive to piperacillin/tazobactam, imipenem, ceftriaxone, cefixime, ciprofloxacin, and gentamicin. Blood culture from dialysis catheter also grew the same organism with the same sensitivities. Dialysis solution culture did not grow anything. His dialysis catheter was removed after hemodialysis and 1 pint of pack cells was transfused. The patient was also started on erythropoietin 4000 unit subcutaneously three times a week. Intravenous iron was not given in view of sepsis. He was treated with piperacillin-tazobactam 2.25 gm 8 hourly for a total of 14 days. He responded to antibiotics and became afebrile after 48 hours. A second right internal jugular nontunneled catheter was inserted after 48 hours of antibiotics. He was discharged on 16 August 2012 with a new right internal jugular temporary catheter with the advice of three times a week hemodialysis. A plan for arteriovenous fistula after two weeks of antibiotics was also made. His repeat blood cultures on day 5 were negative. His anemia responded to erythropoietin and pack red cell transfusion. After completion of course of antibiotics, he was put on intravenous iron sucrose 100 milligram each dialysis for a total of 10 doses. Subsequent laboratory results on day 5 showed hemoglobin of 10 gm, hematocrit of 30%, MCV 82 fl, WBC 5000 u/L, and platelet count of 170000/mm^3^.

## 3. Discussion

 Uremia is associated with alternation in primary host defense system. Neutrophils exhibit impaired chemotaxis, oxidative metabolism, phagocytic activity, degranulation, intracellular killing, and dysregulated programmed cell death. Factors contributing to neutrophil dysfunction include malnutrition, trace element deficiencies, iron overload, impaired glucose metabolism, hyperparathyroidism, dialysis, and uremic retention solutes. These immunologic abnormalities are complicated by the use of immunosuppressive drugs to treat and control underlying diseases and exacerbated by nutritional deficiencies, the dialysis procedure, and the disruption of cutaneous and mucosal barriers to infection [12].

 There are various case reports of AH infections in CKD patients and presentations tend to vary with dialysis modalities. Systemic infections appear to be more common in patients undergoing hemodialysis (HD). On the other hand, peritoneal dialysis (PD) patients generally present with peritonitis. Our case is unique because of the rare AH involvement of hemodialysis catheters. Although there are multiple case reports of peritoneal dialysis catheter, our case is the first that reported hemodialysis catheter related bacteremia. We speculate that there may be some skin carriage of these organisms which led to dissemination of bacteria through the open skin access of the large bore catheters. Alternatively, touch contamination of dialysis catheter by the patient or dialysis nurse after contact with contaminated water supply or dialysis solution could have resulted in this infection. Steroid exposure and the intrinsic low immunologic threshold of dialysis patients may have contributed to the pathogenesis and virulence of the disease. Catheter removal and early initiation of antimicrobials resulted in successful treatment in our case. The different spectrum of disease presentations in HD and PD patients are summarized in Table 1. Furusu and his colleagues reported necrotizing fascitis and gas gangrene due to AH resulting in amputation in a diabetic patient on hemodialysis [13]. Lins and his colleagues reported fatal bacteremia due to myonecrosis and gas gangrene in a patient on hemodialysis and receiving deferoxamine [14]. Generalized cutaneous vasculitis and septicemia were reported by Ramsy in a patient on hemodialysis [15]. There are case reports of endocarditis [16], parapharyngeal soft tissue infection [17], liver abcsess [18], and fatal pneumonia [19, 20] in CKD patients. There is a recent case report catheter related bacteremia by AH [21]. On the other side, there are multiple case reports of peritonitis by AH in patients with peritoneal dialysis [22–32]. 

 Out of 22 cases reported, 14 patients were cured, 7 patients died, and outcome of 1 patient could not be determined. Overall mortality was 33.33%. The infection appeared to be more serious in HD patients as reflected by the worse mortality (4/8, 50%) as compared to peritoneal dialysis (3/13, 23.07%). Uremia, malnutrition, phagocytic dysfunction, and concurrent immunosuppression were identified as potential risk factors for development of AH sepsis. Contamination of dialysis solution and touch contamination may be the potential sources of infections. Prevention, early detection, and treatment are of paramount importance as the mortality is very high (33.33%).

 In conclusion, AH infections do occur in chronic kidney disease. Judging from our literature review, systemic infections occur more frequently in HD patients while localized peritonitis is more common in PD patients. Chronic kidney disease, concurrent immunosuppression, lifestyle factors, and endemicity of AH can predispose to sepsis. Early dialysis catheter removal and initiation of antibiotics can result in successful treatment of dialysis catheter related sepsis by AH. Our case highlighted an interesting and unreported complication of AH infections, which seemingly can produce a wide spectrum of presentation in CKD patients.

## Figures and Tables

**Table 1:** Summary of case reports of aeromonas infections in chronic kidney disease patients.

Number	Reference	Year and journal	Mode of dialysis	Type of infection	Outcome
1	[13]	1997/Nephrology Dialysis Transplantation	Hemodialysis	Necrotizing fasciitis and gas gangrene	Cured
2	[14]	1996/American Journal of Kidney Diseases	Hemodialysis	Myonecrosis, gas gangrene, and bacteremia	Died
3	[15]	1978/Journal of American Medical Association	Hemodialysis	Septicemia/cutaneous Vasculitis	Died
4	[16]	1978/Medicine	Hemodialysis	Endocarditis	Cured
5	[17]	1991/Head Neck	Hemodialysis	Parapharyngeal soft tissue infection	Cured
6	[18]	1991/Kinderärztliche Praxis	Hemodialysis	Liver abscess	Cured
7	[19]	1981/Chest	?	Pneumonia and bacteremia	Died
8	[20]	2001/Internal Medicine	Hemodialysis	Pneumonia	Died
9	[21]	2013/Infection Control Hospital Epidemiology	Hemodialysis	Catheter related bacteremia	?
10	[22]	1983/Gastroenterology and Hepatology	Peritoneal dialysis	Peritonitis	Cured
11	[23]	1984/Pediatric Infectious Disease	Peritoneal dialysis	Peritonitis	Died
12	[24]	1986/Journal of Infection	Peritoneal dialysis kidney transplant	Peritonitis	Cured
13	[25]	1987/Journal of Hospital Infection	Peritoneal dialysis	Peritonitis	Died
14	[26]	1988/Lancet	Peritoneal dialysis	Peritonitis	Cured
15	[27]	1988/Enfermedades Infecciosas y MicrobiologiaClinica	Peritoneal dialysis	Peritonitis	Died
16	[28]	1990/Journal of Infection	Peritoneal dialysis	Peritonitis	Cured
17	[29]	1991/Revista Española de Microbiología Clínica	Peritoneal dialysis	Peritonitis	Cured
18	[30]	1994/Clinical Infectious Disease	Peritoneal dialysis	Peritonitis	Cured
19	[30]	1994/Clinical Infectious Disease	Peritoneal dialysis	Peritonitis	Cured
20	[31]	1995/Clinical Nephrology	Peritoneal dialysis	Peritonitis	Cured
21	[32]	2005/Peritoneal Dialysis International	Peritoneal dialysis	Peritonitis	Cured
22	[32]	2005/Peritoneal Dialysis International	Peritoneal dialysis	Peritonitis	Cured

## Abbreviations

AH: Aeromonas hydrophila
CKD: Chronic kidney disease.
